# The activity of myeloid cell-specific VHH immunotoxins is target-, epitope-, subset- and organ dependent

**DOI:** 10.1038/s41598-017-17948-0

**Published:** 2017-12-20

**Authors:** Christopher Bachran, Matthias Schröder, Lena Conrad, Juan J. Cragnolini, Fikadu G. Tafesse, Laura Helming, Hidde L. Ploegh, Lee Kim Swee

**Affiliations:** 1 BioMed X Innovation Center, Im Neuenheimer Feld, Heidelberg, Germany; 2 Whitehead Institute for Biomedical Research, Cambridge, MA 02142 USA; 3 Merck KGaA, Darmstadt, Germany; 4 Program in Cellular and Molecular Medicine, Boston Children’s Hospital and Harvard Medical School, Boston, MA 02115 USA

## Abstract

The central role of myeloid cells in driving autoimmune diseases and cancer has raised interest in manipulating their function or depleting them for therapeutic benefits. To achieve this, antibodies are used to antagonize differentiation, survival and polarization signals or to kill target cells, for example in the form of antibody-drug conjugates (ADC). The action of ADC *in vivo* can be hard to predict based on target expression pattern alone. The biology of the targeted receptor as well as its interplay with the ADC can have drastic effects on cell apoptosis versus survival. Here we investigated the efficacy of CD11b or Ly-6C/Ly-6G-specific variable fragments of camelid heavy chain-only antibodies (VHH) conjugated to *Pseudomonas* exotoxin A to deplete myeloid cells *in vitro* and *in vivo*. Our data highlight striking differences in cell killing *in vivo*, depending on the cell subset and organs targeted, but not antigen expression level or VHH affinity. We observed striking differences in depletion efficiency of monocytes versus granulocytes in mice. Despite similar binding of Ly-6C/Ly-6G-specific VHH immunotoxin to granulocytes and monocytes, granulocytes were significantly more sensitive than monocytes to immunotoxins treatment. Our results illustrate the need of early, thorough *in vivo* characterization of ADC candidates.

## Introduction

Conventional and engineered antibodies have become indispensable therapeutic tools in the treatment of autoimmune diseases^1^ or cancer^2^. In oncology, they are used to kill cancer cells directly^2^ or deplete cells that limit the anti-tumor response, such as regulatory T cells^3–5^ or macrophages^6^. Antibodies can induce cell death by antagonizing survival signals, by inducing deposition and activation of complement or through antibody-dependent cell-mediated cytotoxicity (ADCC), a mechanism that relies on the activation of innate cells such as natural killer (NK) cells^2^. Although the action of antibodies through ADCC is sufficient to deplete target cells in some cases, second generation therapeutic antibodies are often conjugated through the use of appropriate linkers to a cytotoxic payload in order to increase and control cellular toxicity^7^.

The activity of antibody-drug conjugates (ADC) depends on numerous factors. Besides pharmacodynamic/pharmacokinetic considerations, the efficacy of ADC depends on antibody affinity, on antigen expression levels, turnover and endocytosis, and finally on susceptibility to apoptosis of the targeted cells. Because most cytotoxic payloads require cytoplasmic delivery, the route of endocytosis is likely to have a major impact on cell killing. Furthermore, endocytosis of the target not only depends on intrinsic properties but also on the mode of antigen binding, for example whether crosslinking occurs or not. Therefore, the potential of ADC drugs and their cytoplasmic delivery should be assessed early also in the context of the conjugation methods used.

We previously reported the isolation of a mouse CD11b-specific variable fragments of camelid heavy chain only antibodies (VHH or nanobody)^8^. We now describe two novel VHHs specific for mouse Ly-6C and Ly6G. In order to characterize their ability to kill myeloid cells *in vitro* and *in vivo*, we conjugated *Pseudomonas* exotoxin A domains II, Ib and III (PE38) to the VHHs in a single step using sortase A (SrtA). Our results show that all VHH immunotoxins were active *in vivo*. Furthermore, our data highlight target-, cell-, and organ-dependent activity.

## Results

### VHH16 and VHH21 recognize mouse Ly-6C and Ly-6G

**Figure 1:**
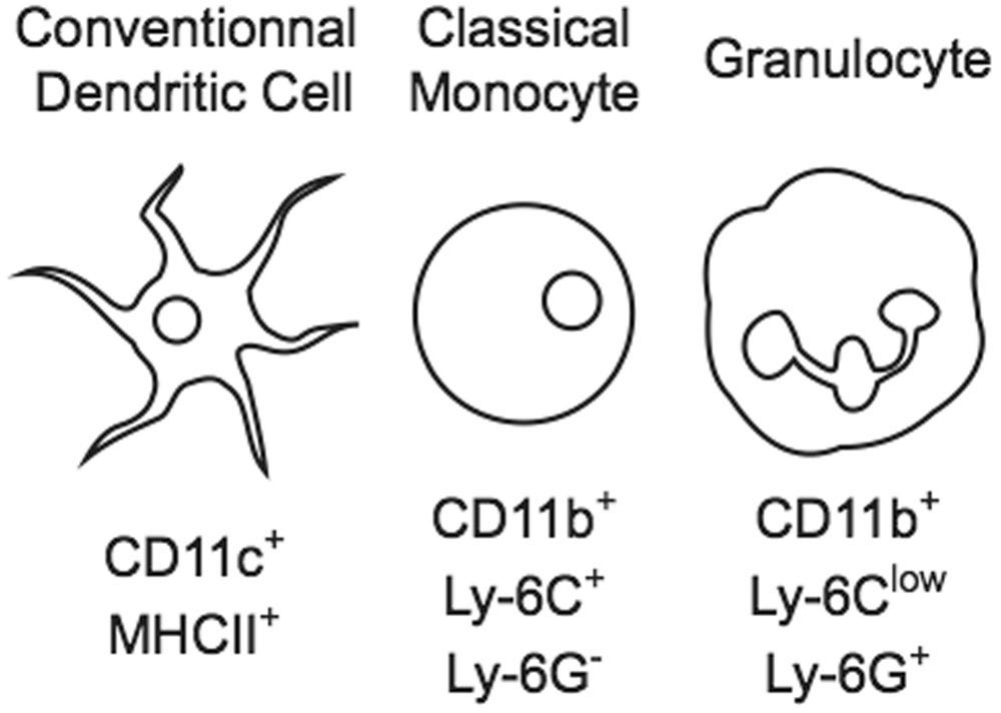
Expression of surface markers by selected mouse myeloid subsets. The scheme shows the expression of CD11c and CD11b integrins, Ly-6C and Ly-6G GPI-anchored proteins and Class II MHC on conventional dendritic cells, classical monocytes and granulocytes.

**Figure 2:**
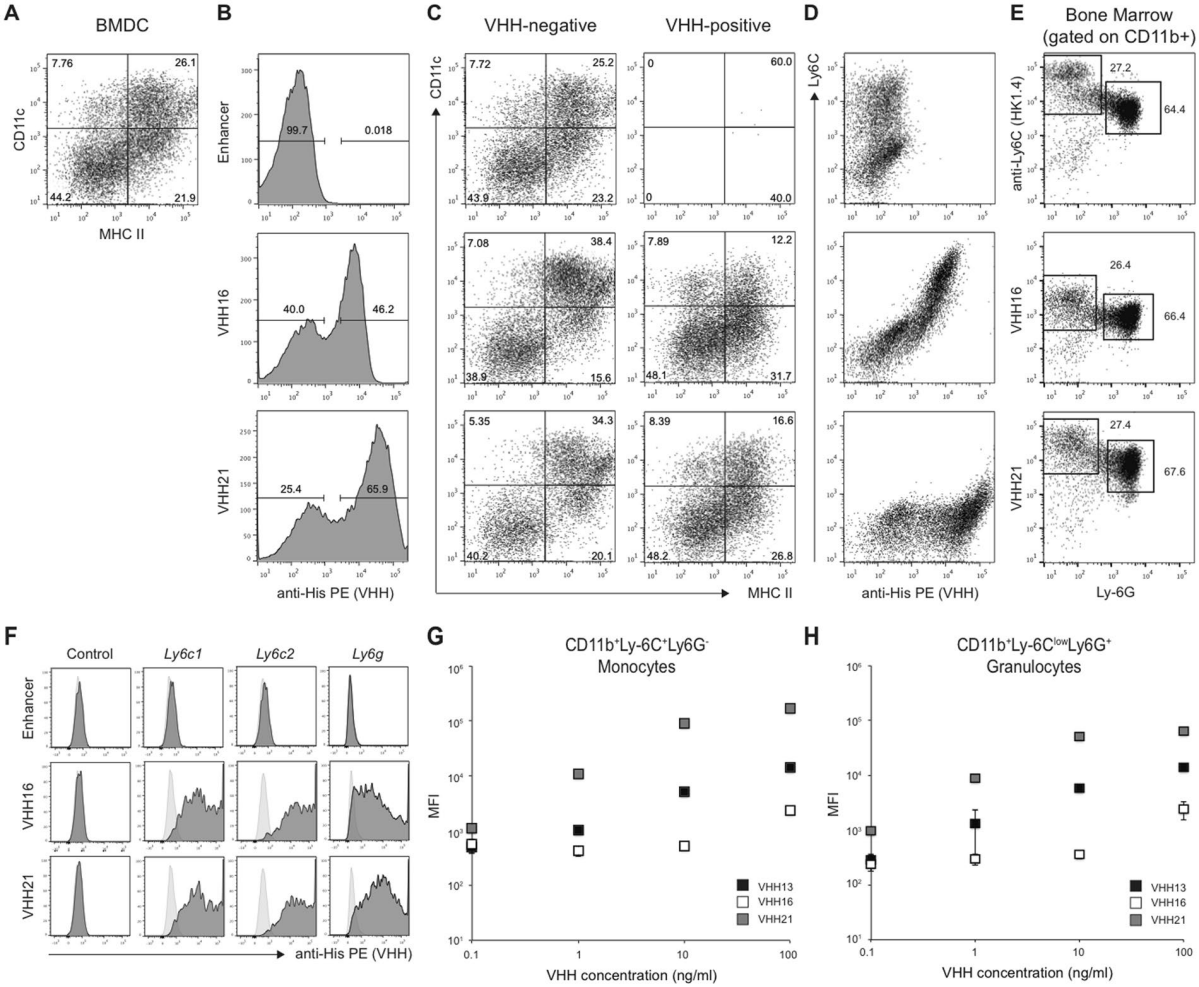
VHH16 and VHH21 recognize mouse Ly-6C and Ly-6G. (**A**–**D**) Mouse BMDC were obtained after differentiation for 6 days with GM-CSF and IL-4. (**A**) MHCII vs CD11c expression on live, differentiated cells. (**B**) Binding of GFP-specific Enhancer, VHH16 and VHH21 on live cells. (**C**) MHCII vs CD11c expression on live cells negative or positive for VHH binding. (**D**) VHH binding vs Ly-6C expression on live cells. (**E**) Ly-6C, VHH16 or VHH21 vs Ly-6G expression gated on CD11b positive fresh bone cells. (**F**) Binding of Enhancer, VHH16 or VHH21 to control HEK 293 cells or cells transfected with *Ly6c1*, *Ly6c2* or *Ly6g* constructs. Light grey histograms: unstained control. (**G** and **H**) Scattered plots show VHHs mean fluorescent intensity (MFI) binding on flow cytometry-sorted monocytic (CD11b^+^Ly-6C^+^Ly-6G^−^) (**G**) or granulocytes (CD11b^+^Ly-6C^low^Ly-6G^+^) (**H**) subsets from fresh bone marrow for indicated VHH concentrations. Each panel representative of 2 independent experiments or more.

Immune cells subsets are characterized by the expression of a specific set of (cell surface) markers. In mice, conventional dendritic cells (DC), classical monocytes and granulocytes can be distinguished by the expression of CD11c and CD11b integrins, Ly-6C and Ly-6G GPI-anchored proteins and Class II MHC (Fig. 1). To isolate VHHs against known or novel antigens, we have previously reported the panning on bone-marrow derived dendritic cells (BMDC) with a phage-displayed VHHs library obtained from the immunization of an alpaca with mouse splenocytes. This strategy yielded VHHs specific for Class II MHC^9^, CD11b (VHH13)^8^ and CD36^10^, while the target of other VHHs (VHH16, VHH21) remained unknown. To identify cells that are recognized by VHH16 and VHH21 we investigated their binding to BMDC. Incubation of fresh bone marrow for 6 days in the presence of GM-CSF and IL-4 led to the differentiation of an appreciable number of cells expressing CD11c as well as MHCII, markers present on mature DC as well as on monocyte-derived macrophages (Fig. 2A)^11–13^. Both VHH16 and VHH21 bound to a sizeable proportion of cells whereas no binding was observed for a GFP-specific VHH (“Enhancer”)^14^ used as a control (Fig. 2B). The comparison of the phenotype (CD11c vs MHCII) of cells negative or positive for VHH-binding suggested that VHHs did not bind to mature CD11c^+^MHCII^+^ cells but rather to CD11c^−/low^MHCII^−/low^ cells, a mixed population that contains common DC precursors, monocyte precursors and monocytes (Fig. 2C). To investigate whether VHHs bound to monocytic cells, we compared the binding of VHH16 and VHH21 to the expression of Ly-6C, a marker highly expressed on monocytes. Binding of VHH16 correlated entirely with Ly-6C expression, whereas VHH21 inhibited anti-Ly-6C binding when co-staining was performed simultaneously (clone HK1.4) (Fig. 2D). Furthermore, the staining of fresh bone marrow with antibodies for CD11b and Ly-6G together with anti-Ly-6C, VHH16 or VHH21 allowed discrimination of monocytic (CD11b^+^Ly-6C^+^Ly-6G^−^) vs granulocytic (CD11b^+^Ly-6C^low^Ly-6G^+^) subsets in identical manner (Fig. 2E). To investigate whether VHH16 and VHH21 bound to Ly-6C (encoded by *Ly6c1* and *Ly6c2*) and Ly-6G (encoded by *Ly6g*), a protein with close sequence similarity, we transfected HEK 293 cells with constructs encoding *Ly6c1*, *Ly6c2* or *Ly6g*. VHH16 and VHH21 but not GFP-specific enhancer bound to cells transfected with *Ly6c1, Ly6c2* and *Ly6g* but not to control, untransfected cells (Fig. 2F). In order to characterize further apparent differences in affinity between VHH16 and VHH21 (Fig. 2B and E), we monitored staining intensity on flow cytometry-sorted monocytic and granulocytic populations incubated with increasing amounts of VHH16, VHH21 and CD11b-specific VHH13 (Fig. 2G and H). The results show that VHH21 bound cells with significantly higher apparent affinity compared to VHH16, where the apparent affinity of VHH13 was intermediate. Therefore, we conclude that VHH16 and VHH21 recognize different epitopes on mouse Ly-6C and Ly-6G and do so with different affinities.

### Engineering and *in vitro* activity of VHH immunotoxins

**Figure 3:**
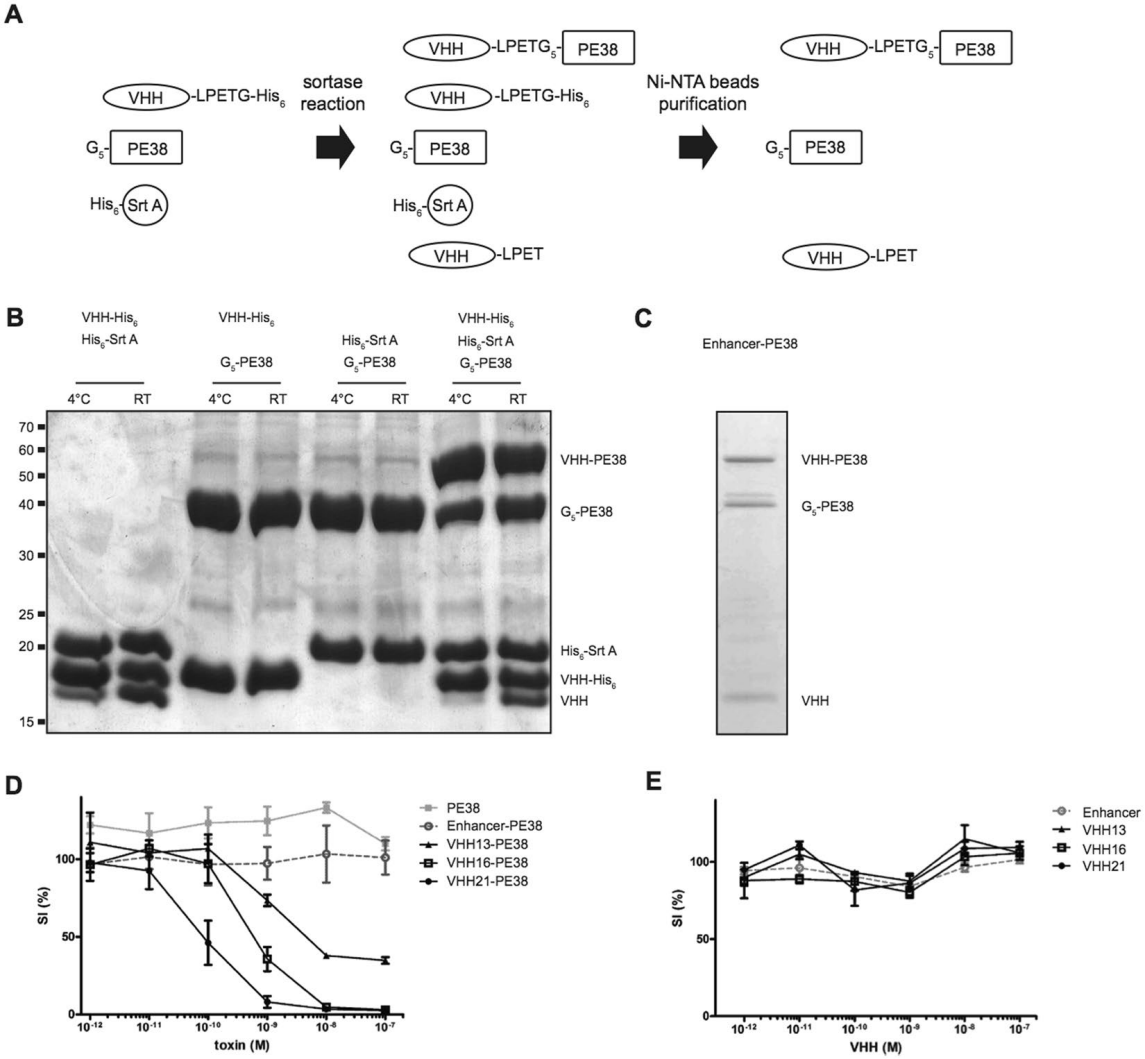
Engineering and *in vitro* activity of VHH-PE38 immunotoxins. (**A**) Schematic representation of SrtA-mediated PE38 conjugation. (**B**) Site-specific conjugation of PE38 to VHH analyzed by Coomassie staining. Reaction was performed at 4 °C or room temperature (RT). (**C**) Ni-NTA purification of conjugation products after SrtA reaction. (**D** and **E**) CD11b^+^Ly-6C^+^ cells were differentiated from NUP98-HOXB4 progenitors and incubated with PE38, VHHs or VHH-PE38 immunotoxins. Cell survival was measured after 48 hours. Representative of 2 independent biological experiments.

In order to investigate the ability of the VHHs to deliver a cytotoxic payload to target cells *in vitro* and *in vivo*, we engineered VHHs fused to *Pseudomonas aeruginosa* exotoxin A. Exotoxin A is composed of 3 domains: domain Ia binds to surface receptors, domain II facilitates membrane translocation, while domain III (and part of domain Ib) ADP-ribosylates elongation factor 2, thereby causing translation arrest and apoptosis^15,16^. Conjugation of domains II, Ib, and III to antibodies can successfully redirect the toxin and induce antigen-specific cell apopotosis^15,16^. SrtA, a cell wall anchoring enzyme of *Staphylococcus aureus*, permits the conjugation of functional groups to proteins bearing a SrtA recognition sequence without the need of supplementary cloning steps or recombinant protein expression^17^. Enhancer, VHH16 and VHH21 were equipped with a SrtA recognition motif followed by a His-tag. We then conjugated PE38 to these VHHs using SrtA, (Fig. 3A). Conjugation of PE38 to VHH occurred equally well at 4 °C or room temperature with an efficiency of 50% or higher (Fig. 3B). Unconjugated (unhydrolyzed) VHH as well as SrtA could be removed in a single step using Ni-NTA agarose beads (Fig. 3C), yielding a final product containing VHH immunotoxin, free toxin and hydrolyzed VHH (Fig. 3A and C). Using this approach, we conjugated PE38 to GFP-specific Enhancer, CD11b-specific VHH13 and Ly-6C-specific VHH16 and VHH21 and investigated their potency *in vitro* and *in vivo*. For this purpose we differentiated mouse bone marrow progenitors immortalized via transduction of NUP98-HOXB4 fusion transcription (“NUP” cells)^18^ factor using GM-CSF and IL-6 for 4 days. This protocol induces the differentiation of a CD11b^+^Ly-6C^+^ monocytic cell population that can be referred to as myeloid-derived suppressor cells^19^. We compared the ability of Enhancer-PE38, VHH13-PE38, VHH16-PE38 or VHH21-PE38 immunotoxins or unconjugated VHH to kill or impede growth of these differentiated cells. VHH13-PE38, VHH16-PE38 and VHH21-PE38 efficiently killed cells, whereas control Enhancer-PE38 had no effect (Fig. 3D). VHH21-PE38 was more potent (SI_50_: 8.9 × 10^−11^ M) than VHH16-PE38 (SI_50_: 6.8 × 10^−10^ M) and VHH13-PE38 (SI_50_: 6.4 × 10^−9^ M). Importantly, free toxin (Fig. 3D) or unconjugated VHHs had no activity (Fig. 3E). Altogether, cell-specific PE38 immunotoxins induced cell death and their potency depends on target and VHH identity.

### *in vivo* activity of VHH immunotoxins

**Figure 4:**
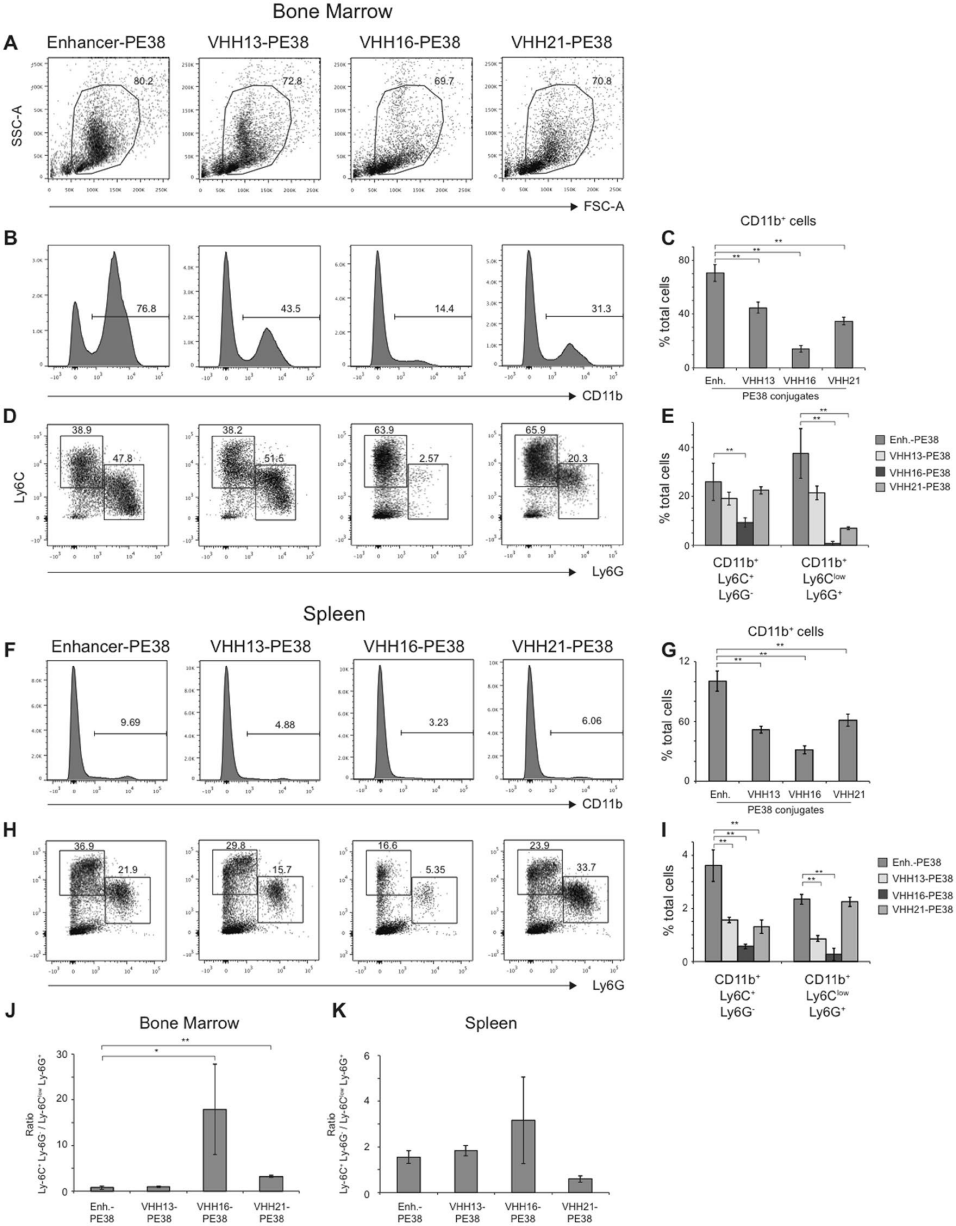
*in vivo* activity of VHH-PE38 immunotoxins. C57BL/6 mice were injected intravenously with (GFP-specific) Enhancer-, VHH13-, VHH16- or VHH21-PE38 immunotoxins. After 24 hours myeloid cell composition was monitored in the spleen and bone marrow. (**A**) Dot plot shows FSC-A vs SSC-A parameters of whole bone marrow of mice injected with indicated immunotoxins. (**B**) CD11b expression on live bone marrow cells. (**C**) Percentage of CD11b^+^ cells in living (SYTOX Blue negative) bone marrow cells. (**D**) Ly-6G vs Ly-6C expression gated on CD11b^+^ live (SYTOX Blue negative) bone marrow cells. (**E**) Percentage of CD11b^+^Ly-6C^+^Ly-6G^−^ and CD11b^+^Ly-6C^low^Ly-6G^+^ in living bone marrow cells. (**F**) CD11b expression on live (SYTOX Blue negative) splenic cells. (**G**) Percentage of CD11b^+^ cells in live (SYTOX Blue negative) splenic cells. (**H**) Ly-6G vs Ly-6C expression gated on CD11b^+^ live (SYTOX Blue negative) splenic cells. (**I**) Percentage of CD11b^+^Ly-6C^+^Ly-6G^−^ and CD11b^+^Ly-6C^low^Ly-6G^+^ in live (SYTOX Blue negative) splenic cells. (**J**) Bar histograms show the CD11b^+^Ly-6C^+^Ly6G^−^/CD11b^+^Ly-6C^low^Ly-6G^+^ cell ratio in the bone marrow. (**K**) Bar histograms show the CD11b^+^Ly-6C^+^Ly6G^−^/CD11b^+^Ly-6C^low^Ly-6G^+^ cell ratio in the spleen. *p value < 0.05, **p value < 0.01 on Student-T test. Representative of 2 independent biological experiments.

We next investigated whether VHH immunotoxins could deplete CD11b or Ly-6C/Ly-6G positive cells *in vivo*. In mice, CD11b is expressed on several myeloid subsets including monocytes, macrophages and granulocytes, Ly-6C is highly expressed by monocyte subsets and lower level on granulocytes, while Ly-6G is expressed by granulocytes^13,20^ (Figs 1 and 2E). We injected VHH immunotoxins intravenously, sacrificed mice 24 hours later and monitored myeloid cell composition in the bone marrow and the spleen. The inspection of bone marrow cells using Forward Scatter (FSC-A) and Side Scatter (SSC-A) parameters showed a striking depletion of SSC-A high cells, presumably granulocytes, in mice injected with VHH16-PE38 (Fig. 4A). The injection of VHH13-PE38, VHH16-PE38 and VHH21-PE38 but not GFP-specific Enhancer-PE38 induced depletion of CD11b^+^ cells in the bone marrow, although to different extents. VHH13-PE38 and VHH21-PE38 induced a moderate reduction of CD11b^+^ cells, whereas injection of VHH16-PE38 induced a 5-fold decrease (Fig. 4B and C). Furthermore, VHH16-PE38 injection induced a 2-fold decrease of CD11b^+^Ly-6C^+^Ly6G^−^ monocytes and nearly completely depleted CD11b^+^Ly-6C^low^Ly6G^+^ granulocytes resulting in an increase in the percentage, but not absolute number of CD11b^+^Ly-6C^+^Ly6G^−^ monocytes within CD11b positive cells (Fig. 4D and E). In contrast, injection of VHH21-PE38 resulted in a decrease of CD11b^+^Ly-6C^low^Ly6G^+^ granulocytes only. In the spleen, depletion of CD11b^+^ positive cells by immunotoxins was similar compared to bone marrow with some notable differences (Fig. 4F and G). VHH13, VHH16 and VHH21 all depleted CD11b^+^Ly-6C^+^Ly6G^−^ monocytes, while only VHH13 and VHH16 depleted CD11b^+^Ly-6C^low^Ly6G^+^ granulocytes (Fig. 4H and I).

**Figure 5:**
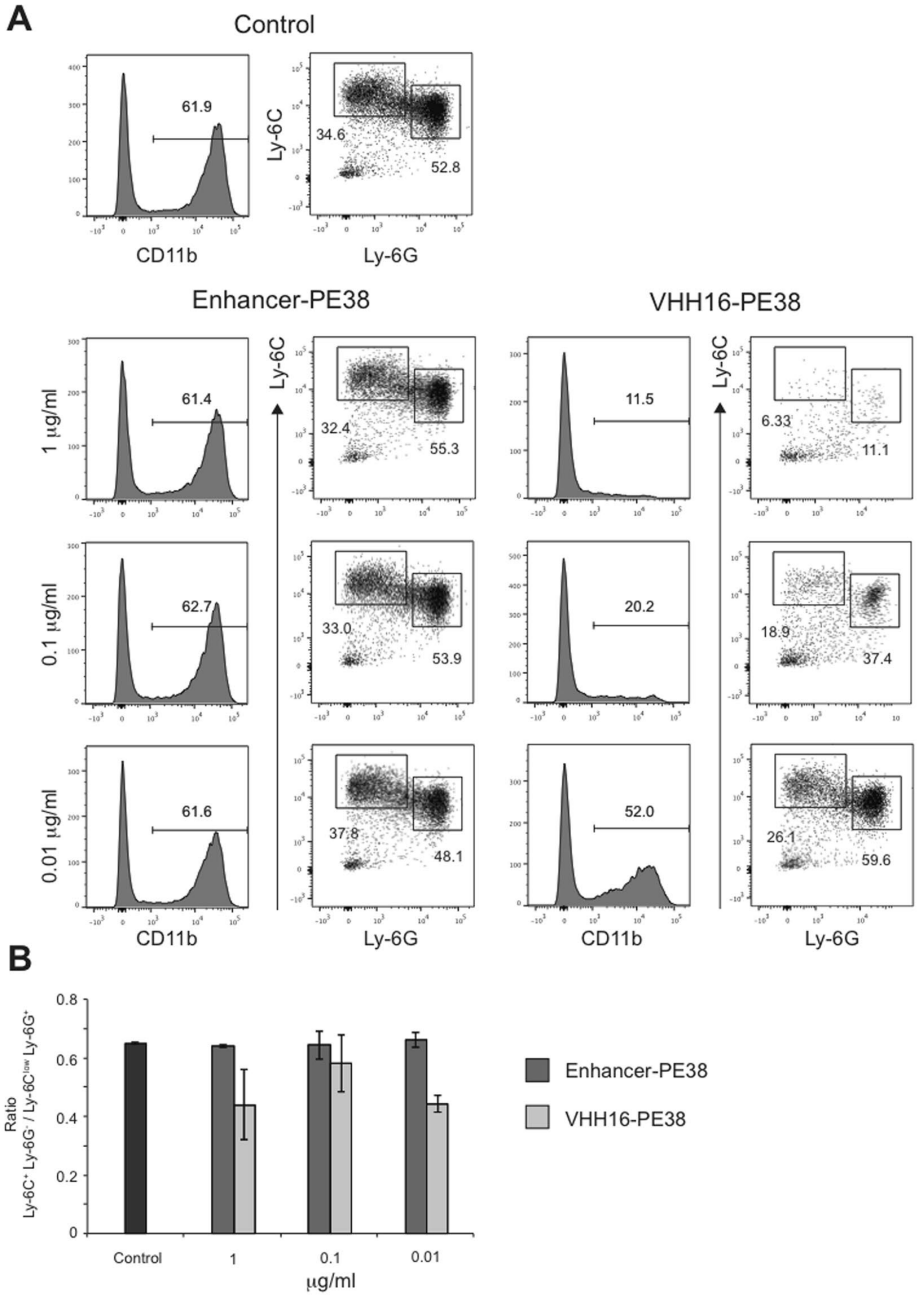
Sensitivity of monocytes and granulocytes to VHH16-PE38 immunotoxin. Fresh bone marrow cells were incubated overnight with different concentrations of (GFP-specific) Enhancer-PE38 or VHH16-PE38. (**A**) Histograms show the percentage of CD11b^+^ cells in living (SYTOX Blue negative) bone marrow cells. Dot plots show Ly-6G vs Ly-6C staining on CD11b^+^ live (SYTOX Blue negative) bone marrow cells. (**B**) Bar histograms show the CD11b^+^Ly-6C^+^Ly6G^−^/CD11b^+^Ly-6C^low^Ly-6G^+^ ratio for indicated conditions. *p value < 0.05, **p value < 0.01 on Student-T test. Representative of 2 independent biological experiments.

Our results show that granulocytes were more sensitive to Ly-6C/Ly6-G-specific immunotoxins as shown by a drastic increase of monocytes to granulocytes ratio (Fig. 4J and K). To investigate whether this effect was due to cell-intrinsic properties such as an increased propensity to apoptosis, we incubated fresh bone marrow cells *in vitro* with decreasing concentrations of Enhancer-PE38 or VHH16-PE38. As expected, VHH16-PE38 depleted CD11b^+^ cells in a dose-dependent fashion (Fig. 5A). However, CD11b^+^Ly-6C^+^Ly-6G^−^ monocytes were equally sensitive to immunotoxins as compared to CD11b^+^Ly-6C^low^Ly-6G^+^ granulocytes, suggesting that differences observed *in vivo* were not cell-intrinsic (Fig. 5A and B).

Altogether, all PE38 immunotoxins were active *in vivo* albeit with remarkable differences depending on target-, cell- and organ identity.

## Discussion

Here we describe the specificity of 2 novel VHHs that recognize different epitopes of mouse Ly-6C and Ly-6G. We have used SrtA to conjugate PE38 to the different VHHs and compared immunotoxin efficacy *in vitro* and *in vivo*. The use of SrtA to effectuate conjugation offers several advantages. First, it avoids cloning and expression of immunotoxins as fusions, not all of which may express and fold equally well. Second, it allows a comparison of the activity of VHHs conjugated to a single batch of recombinant toxin. Third, it avoids potential misfolding caused by difficult to express genetic fusions protein. SrtA has been used to conjugate other toxins such as gelonin^21^, diphtheria toxin and anthrax toxin protective antigen^22^ and is compatible with a variety of proteinaceous and other cargos^17^. However, a potential drawback of the approach is the post-reaction purification procedure that may reduce the number of immunotoxins that can be processed in parallel. For this reason, we have chosen a partial, yet rapid and simple protocol that is compatible with the processing of numerous conjugations simultaneously. Most importantly, we have shown that remaining free PE38 had no measurable toxic activity on cells (Fig. 3).

Although cancer cells have long been the primary target of antibodies or ADC, depletion of B, T and myeloid cells has become an increasingly attractive option for the treatment of autoimmune diseases^23,24^ and cancer^3–6^. Even though the expression pattern of the targeted protein is a major determinant of antibody/ADC specificity, many factors can impact specific killing. In fact, some antibodies can kill specific cell subset despite broader/non-specific target expression. For example, anti-CTLA-4 therapy (Ipilimumab), initially designed to antagonize CTLA-4 on effector T cells, works at least in part by depleting regulatory T cells^3–5^. We have compared here the activity of 3 VHH immunotoxins that recognize different targets (CD11b, Ly-6C, Ly-6G) with overlapping expression patterns on various cell subsets *in vivo*. It allowed us to compare depletion efficiency between VHHs as well as for different cell types and organs, with unexpected findings. First, we demonstrate differences in depletion efficiency between VHH16 and VHH21 that are strikingly dependent on the experimental settings. Although VHH16-PE38 was less potent than VHH21-PE38 *in vitro* (Fig. 3), possibly due to its lower affinity (Fig. 2), the conjugate was significantly more active *in vivo*. These differences could be due to the exact epitope recognized by VHH16 and VHH21, which is known to be different (Fig. 2) or to differences in e.g. immunotoxins half-lives or endocytosis. Second, we find a rather surprising lack of correlation between overall target expression and immunodepletion. Indeed, CD11b^+^Ly-6C^low^Ly-6G^+^ granulocytes were depleted up to 16-fold more efficiently than CD11b^+^Ly-6C^+^Ly-6G^−^ monocytic cells upon treatment with VHH16 and VHH21 (Ly-6C- and Ly-6G-specific) but not VHH13 (CD11b-specific) immunotoxins (Fig. 4E, VHH16-PE38 vs Enhancer-PE38: 37.4% vs 0.8% for CD11b^+^Ly-6C^low^Ly-6G^+^ granulocytes and 25.8% vs 9.2% for CD11b^+^Ly-6C^+^Ly-6G^−^ cells). Third, upon treatment with VHH21-PE38, CD11b^+^Ly-6C^low^Ly-6G^+^ granulocytes were depleted in the bone marrow but not in the spleen. Several hypotheses might explain differences in immunotoxin sensitivity between monocytes and granulocytes. First, there could be cell-intrinsic differences in sensitivity to the immunotoxin, due to differences in the expression of pro- and anti-apoptotic proteins. Second, differences in receptor endocytosis, hence cytoplasmic delivery of immunotoxins, between monocytes and granulocytes could explain differences in killing. Higher rate of Ly-6G endocytosis compared to Ly-6C would have the same consequence, since monocytes do not express Ly-6G. Lastly, granulocytes could be more sensitive to depletion *in vivo* due to increased immunotoxin binding because of enhanced epitope accessibility, anatomical locations or migration patterns. Since monocytic and granulocytic cells were equally sensitive to killing *in vitro* by VHH-PE38 immunotoxins (Fig. 5), we favor the latter hypothesis. Interestingly, other studies have reported significant organ-specific differences in the efficiency of depleting myeloid-derived suppressor cells using anti-Ly-6G (clone 1A8) and an antibody recognizing the Gr-1 antigen common to Ly-6C and Ly-6G (clone RB6-8C5)^25–27^. We conclude that *in vivo* depletion activity of antibodies or their derivatives, such as VHHs, inadequately reflects antigen expression. It is hence a challenge to anticipate potency of immunotoxins, which must therefore be tested empirically *in vivo*. Antibody binding specificity does not necessarily correlate with cell-specific killing *in vivo*. Therefore, pharmacodynamic properties may be more important than antibody specificity.

## Materials and Methods

### Expression and Purification of Recombinant Proteins

VHHs, PE38, and recombinant SrtA^28^ were expressed in *E. coli* strains WK6 (VHH and PE38 expression) and BL21 (DE3) (SrtA expression). Expression cultures in terrific broth (12 g/L tryptone, 24 g/L yeast extract, 5 g/L glycerol, 2.3 g/L KH_2_PO_4_, 12.5 g/L K_2_HPO_4_) in the presence of suitable antibiotics were grown at 37 °C to an OD_600 nm_ of 0.8. Isopropyl β-D-thiogalactopyranoside (BioChemica, #A1008,0025) was added to a final concentration of 1 mM and cultures were incubated for a further 3 hours at 37 °C. Cells were harvested by centrifugation (15 min, 4000 × g, 4 °C), the pellets resuspended in 20 mL PBS (150 mM NaCl, 8.3 mM Na2HPO4, 1.7 mM KH2PO4, pH 7.4)/400 mL culture. Resuspended samples were lysed by sonication (2 × 1 min sonication, 100% intensity, 50% duty cycle, Sonicator HD2070, Sonotrode KE76, Bandelin), centrifuged (30 min, 38000 × g, 4 °C) and the supernatants applied to nickel-nitrilotriacetic acid agarose (Protino Ni-NTA, Macherey-Nagel) columns. All expressed proteins contain a 6 × His-tag, allowing the purification of proteins by metal chelate chromatography. The columns were washed subsequently by PBS, PBS + 20 mM imidazole, and PBS + 50 mM imidazole. Proteins were eluted by PBS + 250 mM imidazole.

The eluted proteins were concentrated in Amicon centrifugal filter devices (Millipore) with a 3 kDa cutoff (for VHHs) and 10 kDa cutoff (for SrtA and PE38). The concentrated proteins were dialyzed overnight against PBS. The concentration of dialyzed proteins was determined by absorbance measurements at 280 nm. The purity of the purified proteins was analyzed by reducing SDS-PAGE (12% gel) and Coomassie staining. The final material was determined to be >90% pure.

### Flow Cytometry

Antibody staining was done in presence of Fc receptor blockade (TruStain fcX BioLegend, #101320) in FACS buffer (PBS with 1% FBS). SytoxBlue (Thermo Fisher Scientific, S34857) was used for exclusion of dead cells. Antibodies used for flow cytometry were as follows (all from BioLegend unless indicated otherwise): anti-Gr1 (clone RB6-8C5), anti-CD11c (clone N418), anti-CD11b (M1/70), anti-MHCII (M5/114.15.2), phycoerythrin (PE)-anti-His (Miltenyi, #130-092-691), anti-F4-80 (clone BM8), anti-Ly6C (clone HK1.4), anti-Ly-6G (clone 1A8). Cells were stained in FACS buffer containing antibodies at 4 °C for 30 min, washed and resuspended in FACS buffer. A FACSAria IIu (BD Biosciences) and FlowJo software (TreeStar) were used for sorting, acquisition and analysis.

### Sortase A-mediated Protein Conjugation

Removal of the N-terminal His-tag and generation of an N-terminal glycine residue on PE38 was achieved by TEV protease cleavage. TEV protease was purified as described in^29^. The gel filtration step described in the protocol was omitted and the pooled fractions of the Ni-NTA-agarose purification were dialyzed (25 mM NaH_2_PO_4_, pH 8.0, 0.1 M NaCl, 10% glycerol, 2 mM EDTA, 10 mM DTT) and frozen in aliquots. The total amount of PE38 recovered after purification from 2.4 L of culture was determined to be ~200 µg. This material was incubated overnight with 20 µg TEV protease (in 50 mM Tris pH 8.0, 0.5 mM EDTA pH 8.0, 1 mM DTT) at 4 °C. The sample was dialyzed against PBS and loaded on Protino Ni-TED 2000 packed columns (Macherey-Nagel, #745120.5). Only the flow-through was collected and the protein concentration determined as described above. Successful cleavage by TEV protease was confirmed by SDS-PAGE.

SrtA-mediated protein conjugation of purified VHHs and TEV protease-cleaved PE38 was performed in 1 × SrtA buffer (50 mM Tris-HCl, pH 7.5, 150 mM NaCl). 50 µM VHH was incubated with 50 µM PE38 and 25 nM SrtA was added. Samples were incubated overnight at 4 °C. Samples were loaded on Protino Ni-TED 2000 packed columns (Macherey-Nagel, #745120.5) to remove unreacted input VHH. Only the flow-through was collected and the protein concentration determined as described above. Successful conjugation by SrtA was confirmed by SDS-PAGE.

### Cell Culture, HEK 293 transfection and Cytotoxicity Assays

Mouse *Ly6c1* and *Lyc2* genes were synthetized and cloned into pCDNA3 expression vector. HEK 293 cells (ATCC, #CRL-1573) were cultured in Dulbecco’s modified Eagle’s medium (Life Technologies, #61965-026) containing 10% fetal bovine serum (Life Technologies, #10270106), 100 U/mL penicillin (Life Technologies, #15140122), and 100 µg/mL streptomycin (Life Technologies, #15140122) in tissue culture flasks at 37 °C in the presence of 5% CO_2_. Cells were transfected using jetPEI Transfection Reagent (Polyplus, #101-10 N) according to manufacturer’s instructions.

RAW 264.7 macrophages (ATCC, #TIB-71) were maintained in Dulbecco’s modified Eagle’s medium (Life Technologies, #61965-026) containing 10% fetal bovine serum (Life Technologies, #10270106), 100 U/mL penicillin (Life Technologies, #15140122), and 100 µg/mL streptomycin (Life Technologies, #15140122) in tissue culture flasks at 37 °C in the presence of 5% CO_2_.

NUP98/HOXB4 cells^18,19^ were cultivated in complete RPMI: RPMI1640 medium (Life Technologies, 21875-034) supplemented with 10% fetal bovine serum (Life Technologies, #10270106), 100 U/mL penicillin (Life Technologies, #15140122), 100 µg/mL streptomycin (Life Technologies, #15140122), 1 mM sodium pyruvate (Life Technologies, #11360070), 2 mM 2-mercaptoethanol (Life Technologies, #31350-010), and 1 × non-essential amino acids (Life Technologies, #11140-035). NUP98/HOXB4 cells were generated from murine bone marrow, isolated from C57/B6 mice and retroviral transduction as described earlier^18,19^. The methods for the isolation of bone marrow from mice were carried out in accordance with the relevant guidelines. All experimental protocols were approved by the Regierungspräsidium Karlsruhe (approved project T55/14). The methods for retroviral transduction of bone marrow cells were carried out in accordance with the relevant guidelines. All experimental protocols were approved by the Regierungspräsidium Tübingen (approved project in facility BIOMEDX.HD.01.02). The complete RPMI medium was furthermore supplemented with 20 ng/mL murine IL-6 (Biolegend, #575708) and 10 ng/mL murine SCF (Biolegend, #579708). Cells were grown in tissue culture flasks at 37 °C in the presence of 5% CO_2_. Every two or three days the NUP98/HOXB4 cells were split to a concentration of 0.2–0.3 × 10^6^ cells/mL for up to three weeks. To induce myeloid differentiation, NUP98/HOXB4 cells were pelleted and resuspended in complete RPMI medium supplemented with 20 ng/mL murine IL-6 and 20 ng/mL murine GM-CSF (Biolegend, #576304).

Dose-response curves for the VHH-PE38 conjugates and for VHHs alone were obtained by incubation on myeloid differentiated NUP98/HOXB4 cells after 4 days of differentiation. 50,000 cells were seeded in 96-well plates in 100 µL myeloid differentiation medium supplemented with the VHH-PE38 conjugates or VHHs alone and incubated at 37 °C for 48 hours in the presence of 5% CO_2_. 20 µL of Cell Titer Blue Cell Viability Assay reagent (Promega, # G8080) was added to each well and the samples were incubated one additional hour before measuring the fluorescence of each well according to the manufacturer’s protocol in a GloMax Multi plate reader (Promega). Survival indices for each sample were calculated in comparison to untreated controls. The 50% survival indices (SI50, 50% cell survival in comparison to untreated controls) values for cytotoxicity analyses were obtained from a nonlinear regression curve fit using GraphPad Prism 5.00. The method used for the nonlinear regression curve fit was “log(inhibitor) versus normalized response” (by using a least square fit).

Cytotoxicity of VHH-PE38 constructs on bone marrow cells was assessed by incubating 200,000 ACK-lysed (Life Technologies, #10492-01) bone marrow cells with immunotoxin in flat bottom 96-well plates in complete RPMI supplemented with 20 ng/mL GM-CSF and 100 ng/mL G-CSF (Biolegend, #574602). Live cell composition was monitored after 20 hours using live-dead staining (SytoxBlue) together with CD11b-, Ly-6C- and Ly-6G-specific antibodies.

### LPS Removal and TNF alpha ELISA

Purified VHH Enhancer-PE38, VHH DC13-PE38, VHH DC16-PE38, and VHH DC21-PE38 were loaded on Pierce High-Capacity Endotoxin Removal Resin columns (Thermo, # 88274) with overnight incubation at 4 °C and eluted by centrifugation (1 min, 500 × g). Protein concentrations were determined as described above.

Analysis of lipopolysaccharide removal was performed by incubation of proteins on RAW 264.7 macrophages. 1 × 10^6^ cells/well were seeded in 2 mL cell culture medium overnight. 0.5 µg protein was incubated on cells in 2 mL fresh medium for 18 hours at 37 °C in the presence of 5% CO_2_. Supernatants (1 mL removed, centrifuged 30 min at 4 °C to remove remaining cells and debris) were analyzed in 1:100 and 1:50 dilutions by murine TNFα ELISA (Biolegend, # 430901) per manufacturer’s protocol.

### Animal Experiments

C57BL/6j mice were maintained under specific pathogen–free conditions in the animal facility of the University of Heidelberg. All animal experiments were done in accordance with German legislation governing animal studies (approved project G-250/14 by the Regierungspräsidium Karlsruhe). Female C57BL/6j mice (8 weeks old, Charles River) were injected intravenously with 10 µg of VHH-PE38 conjugate in 100 µL PBS (Life Technologies, #14190-094). After 24 hours mice were sacrificed, and spleen and bone marrow were isolated. Spleens were mashed (70 µm sieve) in 5 mL complete RPMI medium, centrifuged (5 min, 300 × g), resuspended in 1 mL ACK lysis buffer to remove red cells, incubated 5 min at room temperature, followed by addition of 5 mL complete RPMI1640 medium and re-centrifuged. Cells were resuspended in 5 mL FACS buffer. Bone marrow cells from one femur and one tibia were collected in 5 mL complete RPMI medium, centrifuged and lysed in ACK lysis buffer as described for splenocytes. Bone marrow samples were then resuspended in 1 mL FACS buffer.

### Data Availability Statement

The datasets generated during and analyzed during the current study are available from the corresponding author upon reasonable request.
